# The Impact of Leadership Styles on Quality and Financial Performance in High Medicaid Nursing Homes

**DOI:** 10.1093/geroni/igaa057.2363

**Published:** 2020-12-16

**Authors:** Justin Lord, Akbar Ghiasi, Ganisher Davlyatov, Robert Weech-Maldonado

**Affiliations:** 1 LSU-Shreveport, Shreveport, Louisiana, United States; 2 University of Incarnate Word, San Antonio, Texas, United States; 3 University of Alabama at Birmingham, Birmingham, Alabama, United States; 4 UAB, Birmingham, Alabama, United States

## Abstract

This study examined the association between leadership styles (autocrat, consultative autocrat, consensus manager, and shareholder manager) and resident quality and financial performance in under-resourced nursing homes. Survey data from 391 Directors of Nursing were merged with secondary data from LTCFocus, Area Health Resource File, Medicare Cost Reports, and Nursing Home Compare. Two multivariate regressions were used to model the relationship between leadership styles and the dependent variables: nursing home star ratings (1-5) and operating margin. The independent variables were composite scores for leadership styles, while control variables included organizational and county-level factors. Results show that compared to autocratic leadership, the consultative autocrat (solicits feedback but has total authority) was associated with lower quality (p < 0.05), while the consensus manager (delegates authority to the group) was associated with lower profit margin (p < 0.05). Under-resourced facilities need to recognize trade-offs of different decision making styles for performance.

